# Valorization of Brewer’s Yeast Waste as a Low-Cost Biofiller for Polylactide: Analysis of Processing, Mechanical, and Thermal Properties

**DOI:** 10.3390/ma18215052

**Published:** 2025-11-06

**Authors:** Krzysztof Moraczewski, Małgorzata Łazarska, Magdalena Stepczyńska, Bartłomiej Jagodziński, Tomasz Karasiewicz, Cezary Gozdecki

**Affiliations:** Faculty of Materials Engineering, Kazimierz Wielki University in Bydgoszcz, Chodkiewicza 30 str., 85-064 Bydgoszcz, Poland; m.stepczynska@ukw.edu.pl (M.S.); bar.jag@ukw.edu.pl (B.J.); tomakara@ukw.edu.pl (T.K.); gozdecki@ukw.edu.pl (C.G.)

**Keywords:** brewer’s yeast, polylactide (PLA), industrial waste, polymer composite, circular economy

## Abstract

The aim of this study was the valorization of brewer’s yeast waste as a low-cost, biodegradable filler for polylactide (PLA) and the evaluation of the effect of yeast biomass on the processing, mechanical, thermal properties, and biodegradation of the resulting composites. The materials were prepared using extrusion and injection molding techniques, with the addition of brewer’s yeast (Saccharomyces cerevisiae) in amounts ranging from 5 to 30 wt%. Fourier-transform infrared spectroscopy (FTIR) analysis revealed the absence of strong interfacial chemical interactions, indicating physical dispersion of the filler within the matrix. The addition of biomass significantly modified the properties of PLA. The results demonstrated increased melt flowability (melt flow rate increased from 18.8 to 39.8 g/10 min) and stiffness (a 13% increase in Young’s modulus for 20 wt%), accompanied by a considerable reduction in tensile strength (from 63.2 to 20.2 MPa) and impact strength (from 22.8 to 6.2 kJ/m^2^). Thermal analyses showed a systematic decrease in the glass transition temperature by approximately 5 °C and a dual effect of the filler on crystallization behavior. At low concentrations, the waste acted as a nucleating agent, while at higher loadings it limited crystallinity, leading to an amorphous structure. Thermal stability decreased with increasing biomass content (from 329.3 °C to 266.8 °C). Industrial composting tests indicated that at a 30 wt% yeast content, the mass loss (27.5%) was higher than that of neat PLA (25.5%), suggesting accelerated biodegradation. Despite the deterioration of mechanical performance, the developed biocomposites represent a promising material for single-use applications, combining low cost, easy processability, and an environmentally favorable profile consistent with the principles of the circular economy.

## 1. Introduction

Polymer composites belong to a group of engineering materials that have been developing at a particularly dynamic pace in recent decades. They are used both in advanced industrial sectors and in numerous everyday products. Their essence lies in combining a polymer matrix with an additional reinforcing or filling phase, which enables the achievement of properties exceeding those of the neat polymer. Owing to the wide range of component selection possibilities, such composites can be designed to meet specific requirements from high mechanical strength and resistance to chemical and thermal factors, to the ability to biodegrade under controlled conditions [1,2].

Recently, there has been growing interest in composites produced from biodegradable polymers, such as polylactide (PLA), reinforced with naturally derived components. This approach addresses the need to develop materials aligned with the principles of sustainable development, combining high functionality with minimal adverse environmental impact [3,4,5,6,7].

PLA is one of the most widely used biodegradable polymers, classified as an aliphatic polyester. It is produced either through the ring-opening polymerization of lactide or the condensation of lactic acid, which is obtained from renewable sources such as corn, sugar beet, or sugarcane. This material is characterized by favorable mechanical properties, high stiffness, and transparency, making it competitive with conventional petrochemical-based plastics. Another significant advantage of PLA is its biocompatibility, which enables its use in packaging, additive manufacturing, and medical applications, including the production of implants and surgical sutures. However, the polymer is limited by relatively low thermal resistance and brittleness, which are typically mitigated through chemical modifications or the formation of composites with various natural or synthetic additives [8,9,10,11,12]. Da Silva et al. [10] presented PLA as a biocompatible material with broad applications in implantology and therapeutic systems. They noted that its biodegradation process produces lactic acid, carbon dioxide, and water, which are safely eliminated or metabolized by the body, and that the degradation rate can be adjusted to meet clinical requirements. Swetha et al. [11] conducted a comprehensive analysis of PLA regarding its synthesis, processing, and applications in food packaging. The study discussed PLA production methods, such as lactic acid polymerization, as well as challenges associated with its processing, including material brittleness and difficulties in high-temperature processing. The potential of PLA in food packaging was also examined, emphasizing its biodegradability and role as an eco-friendly alternative to conventional plastics. The authors highlighted the need for further research to enhance PLA properties to enable broader application in this sector.

In recent years, rapid development has been observed in PLA-based composites modified with bio-fillers, aiming to simultaneously reduce production costs, improve performance properties, and increase the share of renewable components within the material structure. The literature extensively reports the use of various plant-derived bio-fillers in polylactide (PLA) composites. The most commonly employed include lignocellulosic fibers, rice husks, wood flour, lignin, and starch, which enable cost reduction and increase the proportion of biodegradable materials in the composite structure [13,14]. Despite their numerous advantages, such fillers often exhibit limited compatibility with the PLA matrix due to differences in phase polarity. This results in poor interfacial adhesion, increased porosity, and reduced thermal stability, ultimately leading to deterioration of properties and processing difficulties [15,16]. To mitigate these unfavorable effects, increasing attention is being directed toward alternative bio-fillers with more homogeneous morphology and controlled chemical composition, sourced from biological waste streams. One such material is brewer’s spent yeast (BSY), characterized by high availability, uniform particle structure, and a balanced biochemical composition. Additionally, the presence of numerous functional groups on the surface of yeast cells enables potential chemical modification to improve interfacial adhesion with the polymer matrix, making yeast an attractive and environmentally friendly bio-filler.

Brewer’s yeast primarily originates from by-products of alcoholic fermentation processes (e.g., beer production) and is subsequently subjected to drying, which allows for long-term storage. It constitutes a valuable feed additive, providing protein, vitamins, minerals, and fiber, while also exhibiting prebiotic and immunomodulatory properties. Beyond their traditional use in animal nutrition, feed yeasts have found wide application in various other industries, including food, cosmetics, and pharmaceuticals [17,18,19].

Yeast biomass can also be utilized as a sustainable component in biocomposites. Owing to its biodegradability and availability as a by-product from the brewing industry, yeast can reinforce polymer matrices such as calcium alginate, bacterial cellulose, or shellac. In biocomposites, it functions as a biosorbent, removing contaminants from water, such as pharmaceuticals or microplastics, improving the mechanical properties of packaging, or serving as a carrier for probiotics in encapsulation processes. Its application contributes to sustainable development by minimizing waste and enabling the production of eco-friendly, functional materials [20,21,22,23,24,25]. In [20] the authors analyzed the biosorption of the antibacterial drug ethacridine lactate (EL) from aqueous solutions using a biocomposite based on yeast biomass immobilized in calcium alginate. The research focused on optimizing process parameters such as pH, biosorbent dosage, initial EL concentration, and contact time, employing the Box–Behnken design within a response surface methodology (RSM) framework. The results demonstrated the potential of low-cost, eco-friendly biocomposites for removing pharmaceuticals from water, supporting the sustainable utilization of brewing industry waste. The aim of the study by other authors [25] was to evaluate the effect of cereal waste (wheat bran and grain processing residues) and brewer’s yeast biomass on the mechanical and barrier properties of wood pulp (NBSK and CTMP). Cereal additives were tested at concentrations of 15% and 40 wt%, while yeast was added at 10 wt%. The results demonstrated that yeast components (proteins and glucans) improved barrier properties (e.g., against water vapor and oxygen) while maintaining or enhancing mechanical performance.

The aim of this study was to analyze the effect of brewer’s yeast biomass addition on the processing, mechanical, and thermal properties of PLA. The novelty of the research lies in the use of a brewing industry by-product as an eco-friendly filler, which has been rarely investigated in the context of PLA, as well as in the detailed characterization of yeast’s influence on the composite’s structure, thermal stability, and durability. Incorporating yeast biomass as a filler in a PLA matrix represents a particularly attractive solution from both technological and environmental perspectives. Yeast is a readily available by-product from the food and biotechnology industries, and its use in composites aligns with the principles of a circular economy. Thus, the developed material combines the intrinsic properties of the biopolymer with an additional ecological benefit derived from the utilization of secondary raw materials. These investigations contribute to the development of new, sustainable biodegradable materials with potential applications in packaging and other environmentally friendly products, integrating circular economy principles with advanced material analysis.

The use of brewer’s yeast biomass as a bio-filler in PLA composites represents a practical implementation of circular economy principles. Employing this biological waste resource enables effective management of post-industrial residues and reduces the consumption of virgin polymer materials. Literature data indicate that incorporating yeast biomass into a PLA matrix can reduce the composite’s carbon footprint by approximately 15–20% compared to neat PLA, while simultaneously lowering raw material costs due to the utilization of a low-value waste feedstock. Such an approach is fully aligned with sustainable development strategies and exemplifies the “waste-to-resource” concept, whereby industrial waste is transformed into value-added raw materials for the fabrication of environmentally friendly polymer composites [26,27].

The study promotes a circular economy by utilizing brewing and cereal industry waste in sustainable food packaging. The developed material should be considered promising, not as a direct substitute for PLA in applications requiring high mechanical strength, but rather as a lower-cost, more sustainable, and easier-to-process alternative for market segments where environmental considerations and the potential for biological recycling are of primary importance.

## 2. Materials and Methods

### 2.1. Materials

The Polylactide (PLA) type 2003D (Cargill Dow LLC, Minnetonka, MN, USA) was selected as the matrix for the new polymer composites. The PLA used had a melt flow rate (MFR) of 6 g/10 min (210 °C, 2.16 kg) and a density (ρ) of 1.24 g/cm^3^.

The filler for the polymer matrix consisted of inactivated brewer’s yeast (Saccharomyces cerevisiae), single-celled microorganisms traditionally used in fermentation processes. The yeast, obtained as a by-product of the brewing industry, is characterized by high contents of proteins, polysaccharides, and trace elements. As reported in the literature [26], the composition of brewer’s yeast typically consists of approximately 45% proteins, about 35% carbohydrates/polysaccharides, and 4–6% ash.

For the study, the brewer’s yeast was added to the polymer matrix in a dried form after preliminary purification and milling to a uniform powder. The preparation process included drying the yeast, mechanical grinding to achieve the desired particle fraction, and homogenization with the polymer during extrusion. The yeast biomass was dried at 60 °C for 24 h to a constant weight prior to incorporation into PLA, and the final moisture content did not exceed 2% (determined gravimetrically). Strict control of water content is crucial to maintain the thermal and mechanical stability of the composites. Average particle size after grinding: approximately 15 µm. The yeast content in the composites ranged from 5 to 30 wt%, depending on the variant studied.

### 2.2. Samples Preparation

The polymeric materials under investigation were produced using two processing methods: extrusion and injection molding. During the extrusion process, granules of the new polymer materials were prepared. Prior to extrusion, the individual components of the polymer blend were mixed in appropriate proportions.

Extrusion was carried out using a 2T20W twin-screw extruder (Łukasiewicz Research Network-Institute of Polymer Materials, Toruń, Poland). The temperatures of the extruder zones and the die were set at 175, 180, 190, and 190 °C, respectively. The extrudate exiting the die was cooled with an air stream and subsequently pelletized.

Samples for testing were then produced from the resulting granulate by injection molding. Injection molding was performed using a TRX 80 Eco screw injection molding machine (Tederic, Taizhou, China) equipped with a mold for standardized 1A paddle-shaped specimens. The sample dimensions were: thickness 4.0 ± 0.2 mm, width of the measuring section 10.0 ± 0.2 mm, length of the measuring section 80.0 ± 0.2 mm, and overall length above 150 mm. The temperatures of the injection molding machine zones were 180, 185, and 185 °C, with a nozzle temperature of 190 °C. The mold was thermostated at 35 °C. The injection and holding pressures were 60 bar and 58 bar, respectively. The cooling time in the mold was 60 s. The final samples were labeled PX, where X denotes the filler content.

### 2.3. Methodology

Infrared spectroscopy (FTIR) measurements were carried out using a Nicolet iS10 spectrometer (Thermo Scientific, Waltham, MA, USA). Fourier-transform infrared spectra (FTIR) were obtained by the attenuated total reflectance method (ATR-FTIR). Each spectrum analyzed was averaged over 16 scans recorded in the wavenumber range from 4000 to 500 cm^−1^.

The density of the materials was determined using a gas pycnometer MVP-D160E (Quantachrome Instruments, Boynton Beach, FL, USA) with helium as the measurement gas. The density value was calculated as the arithmetic mean of three measurements.

Melt flow rate (MFR) tests were performed using an MP600 capillary plastometer (Tinius Olsen, Horsham, PA, USA). Flow rate measurements were conducted at 190 °C under a load of 2.16 kg. MFR values for each material were determined from 12 sample measurements, with the average of 10 measurements reported (the two extreme values were discarded).

Tensile tests were performed on an Instron 3367 universal testing machine (Instron, Norwood, MA, USA) at a crosshead speed of 20 mm/min, using standardized paddle-shaped specimens. Twelve repetitions were performed for each material, and the final values of the measured parameters were calculated as the arithmetic mean.

Surface observations were conducted using an optical microscope VHX7000 (KEYENCE, Mechelen, Belgium) and a scanning electron microscope Phenom XL (Thermo Fisher Scientific, Waltham, MA, USA). SEM images were obtained at a magnification of ×1500, with an accelerating voltage of 10 kV, in mapping mode, using a backscattered electron detector (BSD Full) under high vacuum (0.1 Pa). The fracture surfaces of the samples were coated with a gold layer prior to SEM imaging. The conductive layer was sputtered using a low-vacuum coater MCM100P (SEC) for 60 s.

Impact strength tests (Charpy method) were carried out using an XJ 5Z impact tester (Liangong, Jinan, China) with a 2 J hammer at a drop velocity of 2.9 m/s. Unnotched specimens measuring 80 mm × 10 mm × 4 mm, cut from the tensile test specimens, were tested. The unnotched impact strength (au) was determined, with twelve repetitions per material. The final reported value was the arithmetic mean.

Thermomechanical analysis (DMA) was conducted using a Q800 dynamic mechanical analyzer (TA Instruments, New Castle, WA, USA) over a temperature range of 30–160 °C at a heating rate of 3 °C/min. Specimens were rectangular bars measuring 60 mm × 10 mm × 4 mm, cut from the tensile test specimens. The applied deformation was 15 μm at a frequency of 1 Hz.

Differential scanning calorimetry (DSC) tests were performed using a Q200 instrument (TA Instruments, New Castle, DE, USA) under a nitrogen atmosphere, with a temperature range from 0 to 200 °C and a heating/cooling rate of 10 °C/min. Samples weighing 6.0–7.0 mg underwent three thermal cycles (first heating, cooling, second heating). From the cooling and second heating curves, the glass transition temperature (T_g_), crystallization temperature (T_c_), crystallization enthalpy (ΔH_c_), melting temperature (T_m_), melting enthalpy (ΔH_m_), and degree of crystallinity (X_c_) were determined. The degree of crystallinity (X_c_) was calculated according to Equation (1):

(1) $$X_{c}=\left(\frac{{∆H}_{m}}{{∆H}_{m100\%}\cdot \left(1-x\right)}\right)\cdot 100\%$$

where ΔH_m_100%—the enthalpy of fusion for 100% crystalline PLA; 93 J/g [28]. x—filler content.

Thermogravimetric (TG) analysis was performed using a Q500 thermogravimetric analyzer (TA Instruments, USA) under a nitrogen atmosphere over a temperature range of approximately 25–700 °C, with a heating rate of 10 °C/min. The sample masses ranged from 15.3 to 16.6 mg. From the TG curve, the temperatures corresponding to 5%, 50%, and 95% mass loss (T_5%_, T_50%_, and T_95%_, respectively) were determined, as well as the residue (R) remaining after the test. The T_5%_ value was adopted as a parameter characterizing the thermal resistance (T_d_) of the material. From the derivative thermogravimetric curve (DTG, the first derivative of the TG curve), T_max_ values were also determined, representing the temperatures of the fastest mass loss during each degradation stage.

Biodegradation (organic recycling) under industrial composting conditions was conducted at the Segregation and Composting Plant in Zabrze. Material samples were placed on racks (special baskets designed for this purpose) measuring 27 cm × 70 cm × 21 cm (width × length × height) made of stainless steel. The study was carried out in a designated section of a static compost pile composed of leaves (~40%), wood chips (~30%), and grass (~30%). The pile had a base of 30 m × 33 m and a height of approximately 4 m. The mixed organic waste mass was laid on a naturally aerated platform made of concrete slabs with holes, which allowed for passive aeration of the pile without turning the biomass. Sample baskets were buried at a depth of 1 m below the compost surface. Biodegradation was conducted over 4 weeks at an average temperature of approximately 65 °C. After the designated incubation periods, the baskets were removed from the pile. The samples were then washed with distilled water and dried to a constant mass on filter paper at ambient temperature.

## 3. Results and Discussion

**Figure 1:**
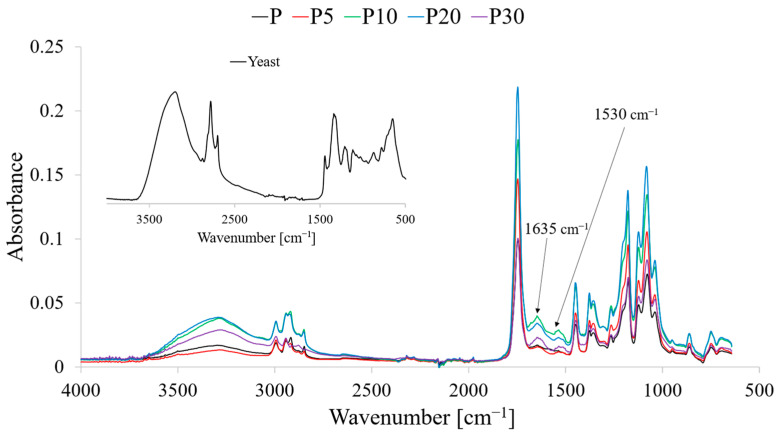
FTIR spectra of the tested materials.

To investigate potential chemical interactions at the matrix–filler interface, Fourier-transform infrared (FTIR) spectroscopy was performed. The obtained spectra for the pure components, the yeast, and the composites are presented in Figure 1.

The FTIR spectrum of neat PLA (P) is dominated by bands characteristic of aliphatic polyesters [23]. The most intense absorption band, with a maximum at 1747 cm^−1^, corresponds to the stretching vibrations of the carbonyl group (C=O) in the ester linkage. Broad and complex bands in the 1300–1000 cm^−1^ region are attributed to the stretching vibrations of C–O–C bonds in the polymer backbone. Bands in the 3000–2900 cm^−1^ range arise from C–H stretching vibrations in methyl groups (–CH_3_).

The spectrum of the filler is typical for a biologically derived material rich in proteins and polysaccharides [29]. A broad band in the 3600–3000 cm^−1^ range, with maximum at 3280 cm^−1^, is associated with O–H stretching vibrations (from water and polysaccharides) and N–H stretching vibrations (from proteins). The broad peak cannot be however associated solely with O–H/N–H stretching vibrations. In this range, significant contributions also arise from asymmetric and symmetric C–H stretching of methyl and methylene groups present in both the PLA matrix and the organic constituents of the yeast. Given the relatively low moisture content (<2 wt%) in the final composites and the overlapping nature of vibrational modes in this region, the increased band intensity observed with rising filler content should be interpreted as a cumulative effect of hydrophilic functional groups and hydrocarbon chains, rather than exclusively hydroxyl- or amine-related stretching. Thus, while the evolution of these spectral features confirms the effective physical incorporation of the yeast phase into the PLA matrix, single-mode assignments must be treated with caution.

Two distinct bands at 1635 cm^−1^ and 1530 cm^−1^ correspond to Amide I (mainly C=O stretching in the peptide bond) and Amide II (a combination of N–H bending and C–N stretching), which are characteristic of protein presence. It should be however noted that the assignment of the 1635 cm^−1^ band exclusively to the Amide I vibration may be complicated by the contribution of residual water in the yeast biomass. The H–OH bending mode of physically and structurally bound water also occurs in this spectral region (approximately 1630–1650 cm^−1^), and although the filler was dried prior to processing, the hydrophilic nature of yeast promotes moisture retention within protein and polysaccharide domains. Therefore, the observed band likely results from the superposition of peptide C=O stretching and water-related bending vibrations, as also reported for other bio-based fillers containing proteinaceous components. In addition, hydrogen-bonding variations and structural heterogeneity of the biomaterials may broaden and shift the Amide I band, further complicating its precise attribution.

The FTIR spectra of the composites exhibit features of both the PLA matrix and the yeast biomass filler. As the yeast content in the material increases (samples P5 to P30), a systematic increase in the intensity of bands characteristic of the filler is observed. This is particularly evident for the Amide II band at 1530 cm^−1^, which is absent in neat PLA, and whose intensity increases proportionally with the yeast content. Similarly, the intensity of the broad O–H/N–H band above 3000 cm^−1^ also increases.

The lack of a visible shifts, together with the absence of any new absorption bands, strongly suggests that interactions at the polymer–filler interface are primarily due to weak van der Waals forces rather than stronger chemical bonds. The inference of predominantly weak (dispersion/van der Waals) interactions rests on several, mutually consistent FTIR observations. First, the carbonyl stretching band of PLA remains centered at ~1747 cm^−1^ across all composites, with no systematic shift to lower wavenumbers beyond the instrument’s typical resolution (≈±2 cm^−1^). In PLA, specific hydrogen bonding to the ester carbonyl usually produces a noticeable red-shift (often ≥5–15 cm^−1^) and/or a resolvable shoulder: neither is observed here. Second, the progressive growth of Amide II (~1530 cm^−1^) and the broad 3600–3000 cm^−1^ envelope scales linearly with yeast loading, consistent with superposition of filler signatures rather than the appearance of new interfacial species. Third, only modest bandwidth/intensity changes are visible for the PLA carbonyl and C–O–C backbone region (1300–1000 cm^−1^) after normalization, with no emergent bands attributable to new covalent linkages. Taken together, these features indicate that the filler is physically dispersed and that interfacial forces, while present, are too weak to produce the spectral fingerprints typical of specific hydrogen-bonded complexes or chemical bonding in PLA-biomass systems. Subtle effects compatible with weak interactions may nevertheless be present: (i) slight line broadening of ν (C=O) without a reproducible frequency shift; (ii) small changes in relative intensity ratios within the C–H stretching region (2990–2940 cm^−1^) after vector normalization; (iii) minor baseline curvature/band tailing in the 3600–3000 cm^−1^ zone due to heterogeneous microenvironments. These nuances support the view that any interfacial interaction is weak and distributed rather than specific and stoichiometric. This FTIR-based interpretation is also congruent with bulk responses (no chemical-interaction signatures in FTIR, reduced T_g_ and damped tan δ peak without new chemical bands), i.e., behavior typical of physical mixing with limited interfacial adhesion.

**Figure 2:**
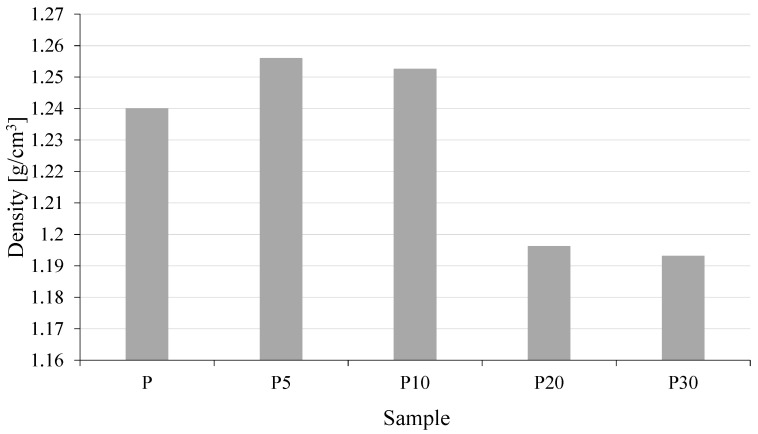
Density of tested materials.

To investigate the effect of waste yeast biomass on the density of the resulting materials, measurements were carried out for the neat polymer (P) and composites containing 5, 10, 20, and 30 wt% filler (P5, P10, P20, and P30, respectively). The obtained results are presented in Figure 2.

Density measurements showed that neat PLA had a value of 1.240 g/cm^3^. The addition of 5 wt% and 10 wt% of waste yeast biomass led to a slight but noticeable increase in density to 1.2559 g/cm^3^ and 1.2525 g/cm^3^, respectively. This effect can be attributed to the filling of free volume in the polymer matrix by the fine biomass particles, resulting in better packing of the system at low dispersed-phase content. In contrast, samples containing 20 wt% and 30 wt% yeast exhibited a decrease in density to 1.1962 g/cm^3^ and 1.1931 g/cm^3^, respectively.

The obtained results indicate a complex influence of yeast biomass on the density of PLA-based composites, which results from both the intrinsic characteristics of the filler and its distribution within the polymer matrix [30]. The nonlinear density behavior arises from a balance between improved packing efficiency at low yeast levels and porosity-driven defect formation at elevated filler contents. Integrating an understanding of the yeast’s surface area, porosity, and hydrophilicity with the mechanical and morphological results provides a more complete interpretation of how biofiller microstructure governs the structural integrity of the developed PLA/yeast composites.

The slight increase in density observed for samples P5 and P10 can be attributed to the more efficient packing of the material, where finely dispersed yeast particles partially occupy free volume between PLA chains and contribute to denser structural arrangement at low filler loadings.

However, at higher concentrations (samples P20–P30), the trend reverses and a noticeable decline in density is observed. This effect is associated with the inherently porous, cellular microstructure of Saccharomyces cerevisiae, which is characterized by considerable surface area and internal voids that are only incompletely infiltrated by the molten polymer during processing. While such morphology is advantageous in biosorption-related applications, in polymer composites it favors the formation of structural discontinuities, including microvoids and entrapped air pockets within agglomerated filler domains. The hydrophilic nature of yeast further contributes to the presence of residual bound moisture and localized polar regions, reducing interfacial wetting and increasing the likelihood of filler pull-out defects.

As a result, the microstructural characteristics of the biofiller—porosity, irregular particle geometry, and agglomeration tendency—become dominant factors controlling the effective density of the composites above 10 wt% yeast content. Therefore, the initial densification effect observed at low filler loading transitions into density reduction at higher concentrations due to the emergence of non-uniform, defect-rich microstructures.

**Figure 3:**
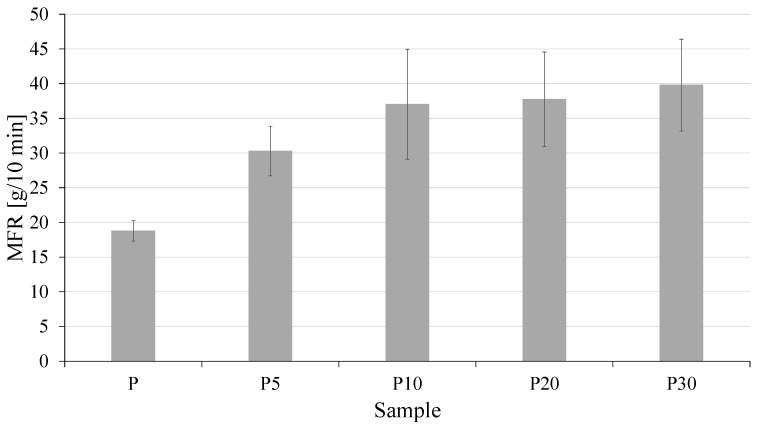
Melt flow rate (MFR) of tested materials.

To assess the effect of yeast biomass addition on the rheological properties of the composites, melt flow rate (MFR) measurements were performed. The obtained results are presented in Figure 3.

The MFR values clearly indicate that the addition of waste yeast biomass to the PLA matrix significantly affects its rheological properties. The MFR of neat PLA was 18.8 g/10 min, whereas for composites containing 5, 10, 20, and 30 wt% biomass, the MFR values increased to 30.3, 37.0, 37.8, and 39.8 g/10 min, respectively. This means that even a small 5 wt% addition of yeast increased the melt flow by over 60% compared to the reference sample, and further increases in yeast content resulted in a gradual, though progressively less pronounced, rise in MFR.

The most commonly reported explanation in the literature for similar phenomena is PLA degradation associated with the presence of moisture and catalytically active substances, which promote hydrolysis of polymer chains during processing. Chain scission leads to a reduction in melt viscosity and, consequently, an increase in MFR. Residual moisture may also have slightly contributed to the reduction in PLA molecular weight and the observed increase in MFR, as a result of hydrolytic degradation of the polymer during extrusion. However, it should be emphasized that the observed increase in flowability can also be interpreted in terms of other mechanisms.

Yeast biomass contains, among other components, lipids, proteins, and polysaccharides [31], which can act as natural lubricants or plasticizers, reducing intermolecular friction and increasing the mobility of polymer segments [32]. The presence of these components may also disrupt the ordered structure of PLA and increase its free volume, further facilitating melt flow. Additionally, yeast particles, acting as a microfiller, can mechanically disturb the polymer entanglement network, thereby lowering the effective viscosity of the system. In light of these considerations, the observed MFR increase should be regarded as the result of a synergistic effect of several factors: partial PLA degradation, the plasticizing and lubricating effects of biomaterial constituents, and the mechanical dispersion of the yeast phase.

**Figure 4:**
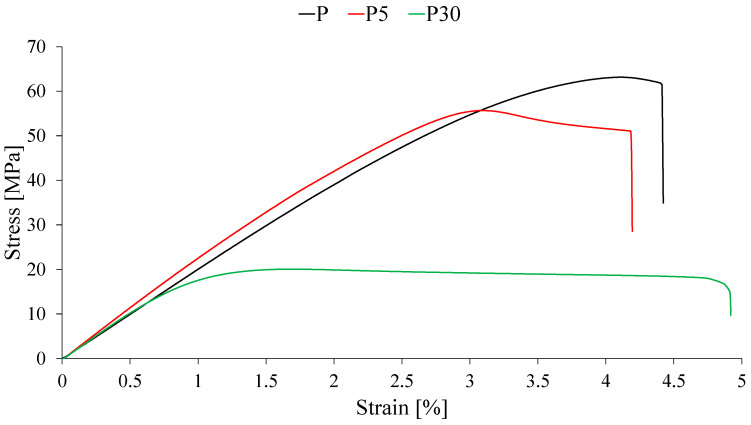
Stress vs. strain curves of the tested materials.

To evaluate the effect of adding waste dead yeast on the mechanical properties of PLA, static tensile tests were conducted on the neat polymer (P) and composites containing 5, 10, 20, and 30 wt% filler (P5, P10, P20, and P30, respectively). The obtained results, including representative stress–strain curves and the values of key mechanical parameters, are presented in Figure 4 and Table 1.

Analysis of the obtained data revealed that the incorporation of waste yeast biomass into the PLA matrix significantly alters the mechanical properties of the resulting biocomposites. A systematic and pronounced decrease in tensile strength (σ_M_) was observed with increasing filler content. Neat PLA exhibited a tensile strength of 63.2 MPa, whereas for the composite containing 30 wt% yeast (P30), this value dropped to 20.2 MPa, representing a reduction of over 68%. Such a substantial decrease in strength indicates poor interfacial adhesion at the matrix–filler interface. This phenomenon is commonly observed in PLA composites with natural, unmodified fillers due to the fundamental chemical incompatibility between the hydrophobic polymer and the hydrophilic surface of most biomasses [33,34]. Instead of reinforcing the matrix through effective load transfer, the filler particles act as stress concentrators, initiating cracks and leading to premature material failure under lower loads [35].

**Figure 5:**
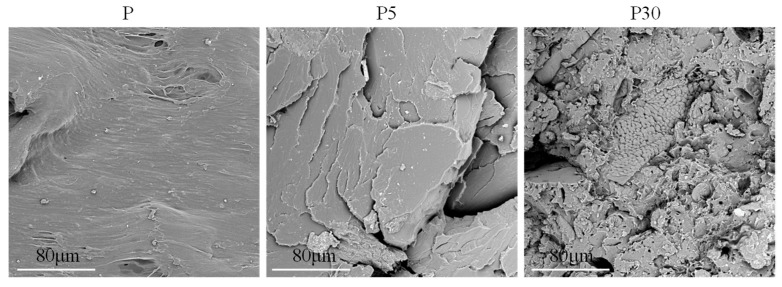
SEM images of the fracture surfaces of selected samples.

The phenomenon of poor interfacial adhesion, which influences the mechanical behavior described in this study, is confirmed by SEM images of the fracture surfaces of the samples, presented in Figure 5.

Analysis of the SEM images of the fracture surfaces revealed a pronounced effect of the addition of waste brewer’s yeast on the composite morphology. The surface of neat PLA (P) was smooth and homogeneous. The incorporation of 5 wt% filler (P5) led to the appearance of isolated, porous yeast agglomerates within the polymer matrix while maintaining relative uniformity and cohesion of the matrix. In contrast, at a high filler content of 30 wt% (P30), significant morphological degradation was observed. The surface became highly rough, with numerous large agglomerates of poorly bonded filler particles, resulting in the formation of numerous cracks and voids, indicative of weak interfacial adhesion.

While particle agglomeration at higher yeast loadings clearly contributes to the deterioration of tensile and impact performance, additional microstructural factors should also be considered. Yeast biomass exhibits a naturally porous cellular architecture, meaning that individual particles contain internal voids that are not infiltrated by the molten polymer during processing. As a result, these particles act not as solid reinforcements but rather as hollow inclusions that reduce the effective load-bearing cross-section of the composite. Moreover, the hydrophilic character of yeast promotes localized moisture retention, which may contribute to interfacial debonding and microvoid formation during cooling, further weakening stress transfer across the matrix–filler boundary. Mechanical properties are undoubtedly also influenced by the crystalline structure of the materials being tested. The relationship between mechanical properties and the crystalline structure of the obtained materials is described later in this article.

In contrast to tensile strength, the Young’s modulus (E), a measure of material stiffness, exhibited an increasing trend. The modulus increased from 2131 MPa for neat PLA to 2408 MPa for sample P20, representing an approximate 13% rise. This is a typical effect of introducing a rigid filler into a polymer matrix, which restricts the mobility of polymer chains and increases the material’s resistance to elastic deformation [36]. However, at the highest filler content (P30), a slight decrease in modulus was observed. This phenomenon can be attributed to the tendency of filler particles to agglomerate at high concentrations. Agglomeration leads to non-uniform dispersion and can introduce structural defects, thereby reducing the effectiveness of the filler in reinforcing the composite [37].

The effect of the filler was also reflected in the change in material ductility, assessed based on the elongation at break (εB). With increasing content of waste yeast biomass, a general decrease in this value was observed, indicating increased brittleness of the composites. The restriction of polymer chain mobility by the filler particles hinders plastic deformation, which is characteristic of particulate composites with poor interfacial adhesion [38].

**Figure 6:**
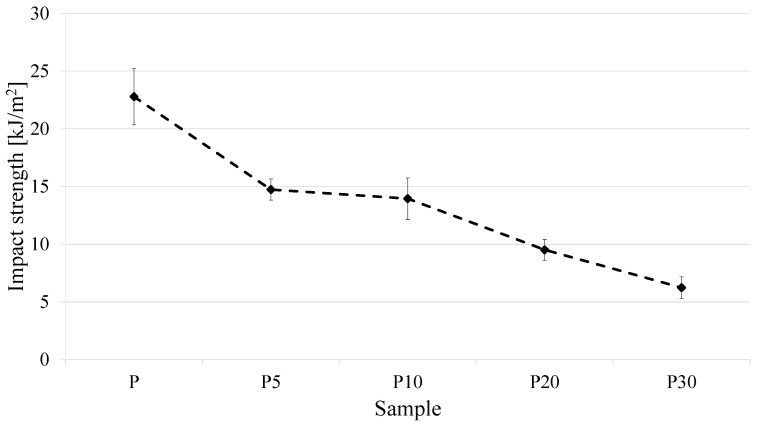
Impact strength of the tested materials.

The obtained impact strength results clearly indicate an adverse effect of the biomass addition on the resistance of PLA to dynamic loading (Figure 6).

Neat PLA (sample P) exhibited an impact strength of 22.8 kJ/m^2^, whereas in composites containing 5, 10, 20, and 30 wt% filler, the values decreased to 14.7, 13.9, 9.5, and 6.2 kJ/m^2^, respectively. This indicates that even at the lowest yeast content, the impact strength dropped by over 35% compared to the reference sample, and at the highest filler loading, the reduction reached approximately 73%. The observed phenomenon can be explained by the nature of the interactions at the PLA–yeast interface. Biomass particles, lacking chemical compatibility with the matrix, act as structural defects and stress concentrators. Consequently, cracks initiate near the filler particles, and their propagation requires less energy than in a homogeneous material. A probable mechanism also involves the formation of microvoids and pores at the interface due to differences in polarity, stiffness, and residual moisture in the biomass, further weakening the structural integrity. Similar trends have been reported in the literature for PLA composites containing other biomaterials, such as plant fibers, cereal husks, or agricultural residues [39], where the lack of filler surface modification resulted in a marked decrease in impact strength. For more specialized applications of the presented polymer material, methods to improve interfacial adhesion, such as yeast surface modification or the use of compatibilizers, will be necessary. Alternatively, reducing the biomass content to a level at which the environmental and cost benefits are achieved without excessive loss of mechanical properties may be considered.

**Figure 7:**
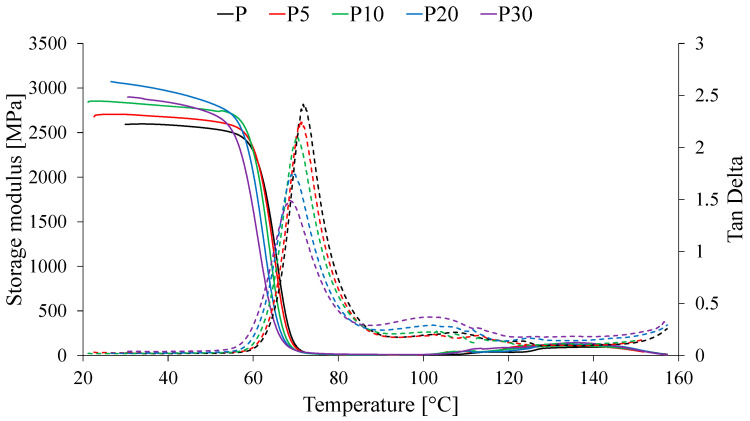
Thermomechanical curves of tested materials.

The effect of adding waste yeast biomass on the thermomechanical properties of PLA was investigated using dynamic mechanical analysis (DMA), and the obtained thermomechanical curves are presented in Figure 7. The measured values of the storage modulus (E′), glass transition temperature (T_gDMA_), and damping factor (tan δ) are summarized in Table 2.

DMA revealed a significant influence of the filler on the temperature-dependent mechanical properties. The storage modulus (E′) in the glassy state (30 °C and 50 °C) systematically increased with yeast content up to 20 wt%. The E′ value for sample P20 was approximately 18% higher than that of neat PLA, which is a typical reinforcement effect in composites, where a rigid filler restricts the mobility of polymer chains within the stiff matrix. However, for sample P30, a slight decrease in the modulus compared to P20 was observed, suggesting the onset of filler particle agglomeration, leading to defects and a reduction in reinforcement efficiency.

Above the thermomechanical glass transition temperature (approximately 80 °C), the storage modulus (E′) of all materials sharply decreases, reflecting the transition to a highly elastic state. Additionally, the thermomechanical curves of the tested materials exhibit an increase in E′ between 100 and 160 °C, attributed to cold crystallization in the PLA matrix, i.e., the secondary ordering of polymer chains. Notably, all composites displayed higher E′ values at 140 °C compared to neat PLA. This behavior can be explained by the formation of interactions between the filler and oriented polymer chains, which restrict chain mobility, as well as a potential network-forming effect induced by components of the waste yeast biomass.

The addition of yeast systematically reduced the thermomechanical glass transition temperature (T_gDMA_), determined from the peak of the damping factor (tan δ), from 71.8 °C for neat PLA to 68.8 °C for the P30 sample. Simultaneously, the amplitude of the tan δ peak was noticeably dampened with increasing filler content. The decrease in T_gDMA_ may indicate weak interfacial adhesion between the polymer matrix and the filler or the plasticizing effect of certain components of the waste yeast biomass. The reduction in tan δ values directly demonstrates a diminished capacity of the polymer chains to dissipate energy in the presence of filler particles, confirming the reinforcing effect and the decrease in the viscoelasticity of the material.

**Figure 8:**
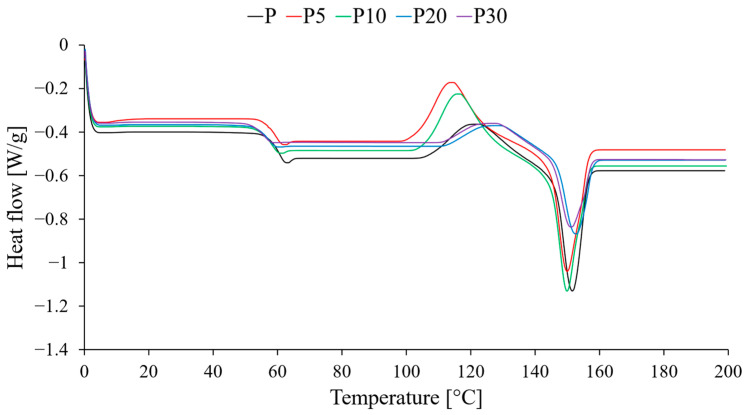
Thermal curves of tested materials.

Thermal properties of the PLA/yeast composites were analyzed using differential scanning calorimetry (DSC) to gain insight into structural changes induced by the filler. The thermograms obtained during the second heating cycle, representing the intrinsic material properties after eliminating thermal history effects, are shown in Figure 8, while the extracted thermal parameters are summarized in Table 3.

DSC analysis revealed a significant effect of the filler on the morphology and thermal transitions of PLA. A systematic decrease in the glass transition temperature (T_g_) was observed, declining from 60.0 °C for pure PLA to 55.6 °C for the composite containing 30% filler. This phenomenon, consistent with DMA results, may be attributed to the presence of low-molecular-weight components in the yeast acting as a plasticizing agent, thereby increasing the mobility of polymer chains, or it may indicate weak interactions at the matrix–filler interface, leading to an increase in free volume and a consequent reduction in compressive stresses.

The addition of the filler also influenced the crystallization process. The cold crystallization temperature (T_cc_) initially decreased from 121.2 °C (P) to 113.4 °C (P5) and subsequently increased at higher filler concentrations. The decrease in T_cc_ at low content (5 wt%) suggests that yeast particles may act as heterogeneous nucleation sites, facilitating crystal formation at lower temperatures. However, the increase in T_cc_ at higher concentrations indicates that an excess of particles poses a physical barrier, hindering the diffusion and ordering of polymer chains, thereby inhibiting crystal growth and requiring higher energy to initiate the process. This behavior is a typical manifestation of the confinement effect at high filler loadings, where aggregates of yeast particles physically obstruct the reorganization of polymer chains, thus suppressing the crystallization process.

The melting temperature (T_m_) did not exhibit significant changes. T_m_ remained relatively constant for all investigated materials, indicating that the addition of yeast does not affect the thermal stability of PLA crystals and, consequently, the filler did not alter their structure or perfection. In contrast, changes in the melting enthalpy (ΔH_m_) were observed. The nature of these changes followed the trend noted for cold crystallization, reaching higher values for samples P5 and P10, and lower values for P20 and P30. The observed changes in the melting enthalpy (ΔH_m_) exhibit a strong correlation with the position and characteristics of the cold-crystallization peak (T_cc_), which is directly related to the crystallization behavior of the PLA/yeast composites. The amount of material melting above T_cc_ is governed by the extent of crystallization occurring between T_g_ and Tm, meaning that ΔH_m_ reflects the effectiveness of this process. At low filler contents (5–10 wt%), the decrease in T_cc_ indicates a heterogeneous nucleating effect of yeast particles combined with enhanced PLA chain mobility, promoting more intensive crystal formation and resulting in higher ΔH_m_ values. In contrast, the increase in T_cc_ at higher filler concentrations (≥20 wt%) suggests that the crystallization kinetics are hindered due to particle agglomeration and restricted chain diffusion, which reduces the effective supercooling and narrows the available crystallization window. As a result, a smaller fraction of the crystalline phase is formed, directly leading to a reduction in ΔH_m_. Thus, the parallel trends in T_cc_ and ΔH_m_ confirm that modifications to the kinetics of cold crystallization are the principal factor governing the morphological structure of the developed biocomposites.

The final observation concerns the effect of the filler on the overall degree of crystallinity (X_c_) of the investigated materials. The calculated values, based on the melting enthalpy and cold crystallization enthalpy, were close to zero for all composites, compared to 1.8% for pure PLA. This indicates that, despite its nucleating potential, the presence of waste yeast biomass in the PLA matrix effectively suppresses the overall crystallization process during cooling from the melt. As a result, the obtained composite materials exhibit a predominantly amorphous structure under practical conditions. This observation is consistent with the mechanical testing results. Low crystallinity, combined with poor interfacial adhesion and a plasticizing effect, accounts for the significant reduction in the mechanical strength of the composites.

Mechanical properties and crystallization behavior in the investigated PLA/yeast composites are closely interrelated, and the observed thermal transitions provide valuable insight into the mechanical performance. The decrease in T_cc_ and the accompanying increase in ΔH_m_ at low filler contents (5–10 wt%) suggest that limited cold crystallization occurs more efficiently due to a mild nucleating effect of yeast particles, leading to a slightly higher fraction of ordered domains that contribute to matrix stiffening. This is consistent with the increase in Young’s modulus observed for samples P5 and P10, confirming that the crystalline phase, even at low absolute levels, locally restricts polymer chain mobility and enhances elastic response. Conversely, at higher yeast concentrations (≥20 wt%), T_cc_ shifts to higher temperatures while both ΔH_m_ and the total degree of crystallinity decrease, indicating significant suppression of crystal growth attributed to particle agglomeration and interfacial incompatibility. This strong inhibition of chain ordering, combined with extensive interfacial defects identified in SEM observations, aligns directly with the pronounced deterioration of tensile and impact strength, as the lack of an effective reinforcing crystalline network and the presence of stress concentrators facilitate brittle fracture. Taken together, the thermal and mechanical data demonstrate that reduced crystallization capacity—particularly in composites with 20–30 wt% yeast—plays a crucial role in weakening the structural integrity of the material, amplifying the negative effects of poor adhesion at the polymer–filler interface.

**Figure 9:**
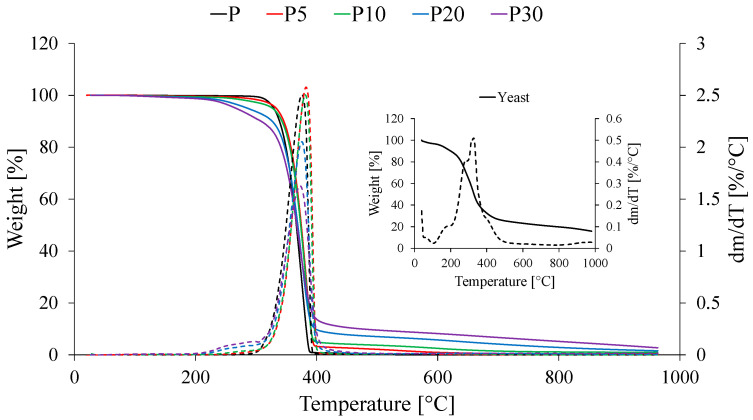
Thermogravimetric curves of tested materials.

The thermal stability of neat PLA and the composites with yeast waste was investigated using thermogravimetric analysis (TGA). The obtained mass loss curves (TGA) and their derivatives (DTG) are presented in Figure 9, while the determined thermal parameters are summarized in Table 3.

Neat PLA (sample P) exhibited a characteristic single-step degradation profile typical of this polymer, with a 5% mass loss temperature (T_d_) of 329.3 °C and a maximum decomposition rate at 376.1 °C. The process proceeds almost completely, leaving negligible residual mass. In contrast, pure yeast, as a biological material, displays significantly lower thermal stability and undergoes a complex, multi-step degradation starting at temperatures below 200 °C. In the first stage, below 150 °C, the release of moisture and volatile compounds occurs present in yeast biomass. Subsequently, in the range of 200–350 °C, the degradation of the main organic constituents is observed, primarily proteins and polysaccharides. In the third stage, above 400 °C, a mineral residue in the form of ash remains.

The incorporation of waste yeast biomass into the PLA matrix generally reduced the thermal stability of the composites, with this effect being particularly pronounced at higher filler concentrations. The onset degradation temperature (T_d_) decreased from over 327.0 °C for samples P and P10 to 286.0 °C for P20 and only 266.8 °C for P30. This reduction in stability is a direct consequence of introducing a thermally less stable component (as confirmed by the analyses performed on the yeast biomass), which initiates the degradation process at a lower temperature. Notably, sample P5 exhibited a slight increase in the onset degradation temperature (T_d_ = 333.3 °C) and the temperature of maximum decomposition rate (T_max_ = 382.7 °C) compared to pure PLA. This phenomenon can be attributed to possible polymer–filler interfacial interactions, which slightly hinder the initiation of the degradation process. Localized interfacial interactions may occur between the functional groups of PLA and the yeast biomass, which is consistent with observations reported for other PLA composites containing bio-fillers [30,39].

Moreover, the addition of the filler altered the degradation mechanism from a single-step to a two-step process, which is clearly visible in the DTG curves of samples P20 and P30. The first, low-temperature peak (around 270 °C) corresponds to the thermal degradation of the yeast biomass components. The second, dominant peak (approximately 375–380 °C) is associated with the main degradation stage of the PLA chains. Its position did not change significantly compared to neat PLA, suggesting that the yeast and its constituents do not accelerate the degradation of the PLA matrix itself but instead introduce an additional, earlier degradation step.

As expected, the amount of residual char after analysis of the yeast-containing materials changed with filler content. The residue after pyrolysis (R) systematically increased with increasing filler concentration, from 0.4% for P5 to 2.8% for P30. This confirms the presence of non-combustible inorganic components (likely mineral salts) introduced with the yeast and is consistent with the theoretical composition of the studied mixtures.

From a practical perspective, the significant reduction in the onset degradation temperature narrows the processing window of the composites. The results indicate that processing composites with high yeast content (≥20 wt%) will require lower processing temperatures to prevent thermal degradation. Therefore, precise temperature control may be necessary during processes such as extrusion or injection molding to avoid premature thermal decomposition and deterioration of material properties. On the other hand, the reduced thermal stability may correlate with increased susceptibility to hydrolysis and biodegradation, which is a highly desirable feature for materials intended for environmentally friendly applications.

**Figure 10:**
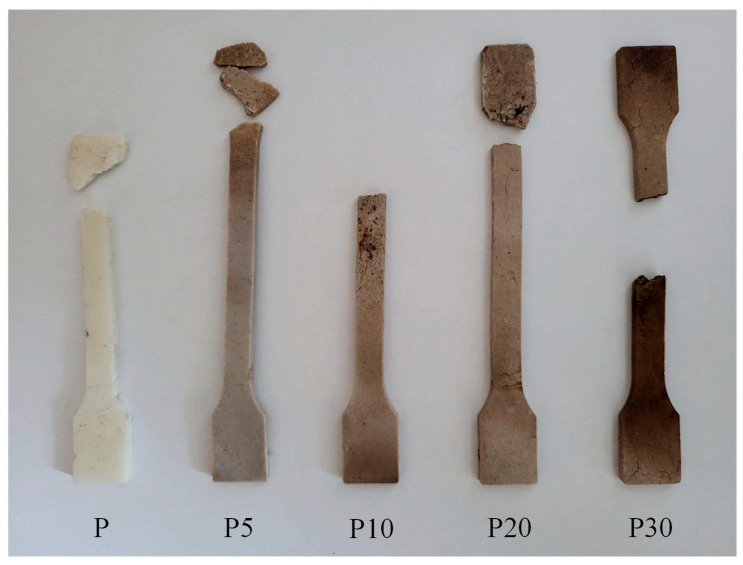
Effects of industrial composting on tested materials.

**Figure 11:**
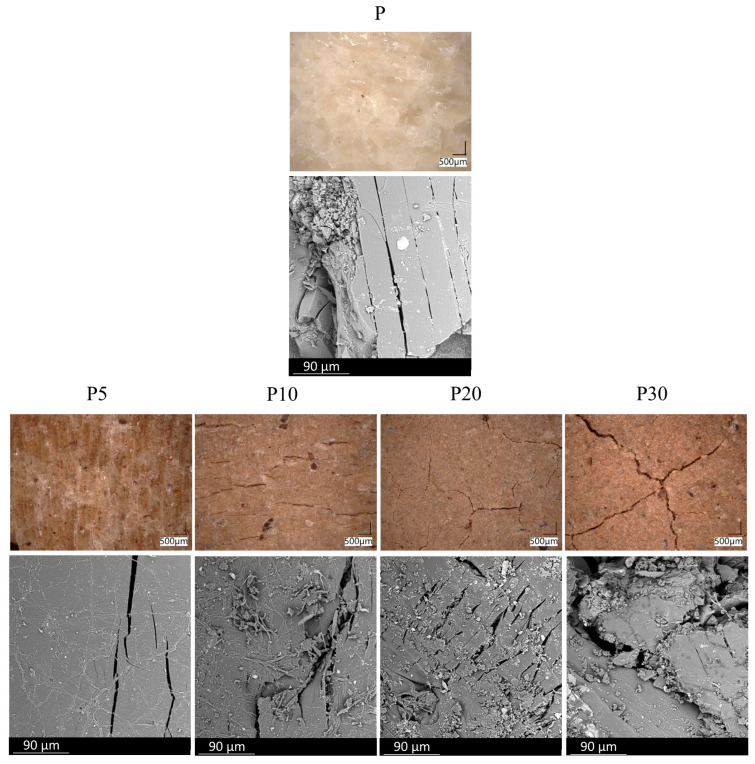
Optical and scanning electron microscopy images of tested materials after industrial composting.

To assess the effect of the applied filler on the biodegradation of PLA, industrial composting tests were conducted on the prepared composites. Photographs and microscopic images of the samples after 4 weeks in the compost pile are presented in Figure 10 and Figure 11.

Visual and microscopic examination of the samples after composting revealed significant differences in the degree of material degradation depending on the sample variant. Sample P exhibited pronounced surface discontinuity, with visible cracks, mattification, and partial disintegration. Initially, the incorporation of the filler into the PLA matrix appeared to inhibit the biodegradation process. Sample P5 showed only minimal signs of degradation, manifested as slight surface erosion, without substantial loss of structural integrity. However, as the filler content increased, the effects of composting became more pronounced. Sample P10 exhibited moderate changes, including partial deformations and localized crumbling. The strongest degradation effects were observed in samples P20 and P30, which displayed deep cracks, loss of cohesion, darkening of the material, and fragmentation into smaller pieces.

**Figure 12:**
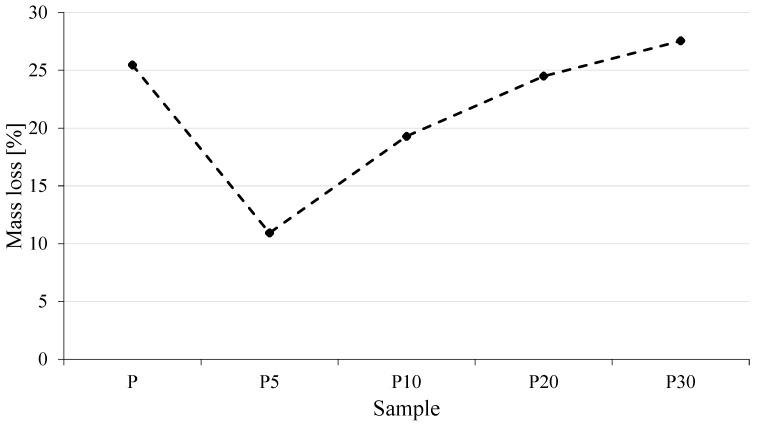
Mass loss of tested materials after industrial composting.

Mass loss measurements after industrial composting conditions (Figure 12) are consistent with the observed visual changes.

Mass measurements indicated that all analyzed samples underwent biodegradation; however, the rate of this process depended on the yeast biomass content. Pure PLA exhibited a mass loss of 25.5% over the test period. The incorporation of 5 wt% waste yeast biomass (P5) significantly reduced the degradation rate, with a mass loss of only 10.9%. This effect can be attributed to the formation of a more compact material structure, in which the biomass particles may have restricted the access of microorganisms and moisture to the polymer matrix.

With further increases in biomass content (samples P10–P30), a gradual rise in the susceptibility of the composites to biodegradation was observed. For samples P10 and P20, mass losses were 19.3% and 24.5%, respectively, while P30 exhibited the highest mass loss of 27.5%, exceeding even that of the reference PLA. This indicates that at high levels of waste yeast biomass, the material becomes more prone to degradation in a composting environment. This effect is likely due to two interrelated factors: first, the higher biomass content promotes microbial activity, which can more effectively degrade the polymer matrix; second, the addition of large amounts of yeast leads to the formation of microporosity and aggregates, facilitating the penetration of environmental factors into the interior of the composite.

Although the yeast biomass used in this study was rendered biologically inactive, its presence could still indirectly contribute to the intensification of PLA degradation. This may be related to the partial stability of certain enzymes retained within the yeast cell structures (e.g., esterases and proteases), which could, to a limited extent, catalyze the cleavage of ester bonds in the polymer chains. Studies on yeast residues indicate that such material may contain active or thermostable hydrolases, or may act as a source of these enzymes under conditions favoring their extraction or reactivation [26].

The hydrophilic nature of the yeast biomass promotes water absorption, which intensifies the hydrolysis of ester bonds in PLA chains. Water introduced into the system, or entrapped within the porous structure of yeast particles, may catalyze polymer degradation by lowering its molecular weight and accelerating the decomposition process. A similar degradation mechanism for polylactide in the presence of biological environments was described by Tsuji and Suzuyoshi [40], who demonstrated that the process proceeds in two stages, involving initial hydrolysis of ester linkages followed by further enzymatic degradation initiated by microorganisms. In the context of this study, yeast biomass could similarly accelerate PLA degradation. Additionally, the hydrophilic character of yeast biomass facilitates water uptake and the formation of localized regions with elevated moisture content, which enhances the hydrolysis of ester bonds in PLA chains, leading to reduced molecular weight and faster degradation [41].

When benchmarked against other PLA–biofiller systems, the PLA/yeast composites display a property profile that is typical in trend but distinctive in magnitude and processing response. For lignin-filled PLA, numerous studies report that low additions (≤5–6 wt%) can maintain or slightly improve stiffness while higher loadings generally depress tensile strength due to interfacial incompatibility, behavior closely mirroring the present yeast system, although lignin can sometimes deliver modest strength plateaus at very low contents depending on dispersion and chemistry [42,43,44]. Wood-flour and other lignocellulosic fillers frequently raise modulus but induce pronounced drops in tensile strength and impact resistance at ≥10–20 wt%, with reported strengths in the ~28–50 MPa range depending on processing (injection- vs. compression-molded/AM), broadly comparable to or slightly higher than your P20–P30 results [45,46]. Starch-containing PLA blends/composites likewise tend to lose strength and toughness unless compatibilized, while their crystallization behavior is highly formulation-dependent [47]. By contrast, agri-waste particulates such as spent coffee fractions commonly produce (i) a viscosity/MFR increase (beneficial for flow) and (ii) stiffness gains with concurrent strength/impact penalties—patterns closely aligned with the yeast outcomes here [48,49]. With respect to thermomechanical response, the small T_g_ decreases and the nucleation–suppression crossovers in T_cc_ observed (lower T_cc_ at low filler; higher T_cc_ and reduced ΔH_m_ at high filler) are consistent with literature on PLA modified by bio-derived particulates and nucleants, where heterogeneous nucleation at low contents can give way to diffusion-limited crystallization at higher loadings.

Overall, yeast positions competitively among low-cost biofillers: it provides a similar stiffness gain window to lignocellulosics and lignin at modest loadings, exhibits the common trade-off of reduced strength/impact at higher loadings, and—distinctively—delivers a substantial MFR increase that can aid processability, together with end-of-life advantages (biogenic origin, compostability synergy). In application niches prioritizing flow, cost, and circularity over high durability (e.g., rigid/semi-rigid single-use packaging), this balance can be advantageous relative to other biofillers that do not improve flow or biodegradation pathways to the same extent [50].

In future studies, to improve the interfacial adhesion of the yeast biofiller to the PLA matrix and thereby better maintain tensile and impact strength at higher filler loadings, several surface treatment and compatibilisation strategies merit consideration. For example, in analogous PLA/wood-flour systems the use of silane coupling agents (e.g., KH-550) substantially improved compatibility [51,52]. Similarly, coupling agents have proven effective in improving filler–polymer interaction in natural-fibre composites. Reactive compatibilisers such as maleated or glycidyl-methacrylate-grafted PLA (PLA-g-MA or PLA-g-GMA) offer another viable route; functional groups react with hydroxyl or amino groups on the filler surface during melt mixing, promoting covalent or ionic bonding and improved stress transfer. In the context of biochar-filled PLA, low filler loadings produced notable modulus gains, but at higher loadings performance declined due to poor dispersion and weak interface [53,54,55]. In our system, the yeast biomass presents both hydrophilic surface groups and internal porosity, which likely hinder effective polymer wetting and create voids; accordingly, a combined approach—mild surface hydrophobisation (e.g., acetylation or fatty-acyl esterification) plus a reactive compatibiliser—could reduce moisture uptake, improve wetting, and restore load-transfer efficiency. The use of a “grafting-from” strategy (e.g., PLA brushes initiated from filler surface –OH groups) may further enhance interfacial continuity, although complexity and cost must be balanced against the benefits in low-cost, high-volume packaging applications. By tailoring filler surface chemistry in this way, the common trade-off between increased stiffness and decreased strength/toughness in PLA bio-composites could be better mitigated, thereby improving both processability and end-use performance.

## 4. Conclusions

In this study, novel biocomposites based on polylactic (PLA) were produced and subjected to comprehensive characterization, using waste yeast biomass as a filler at concentrations ranging from 5 to 30 wt%.

The addition of yeast waste significantly modified the material properties of PLA. FTIR analysis indicated the absence of strong chemical interactions at the interface, suggesting that the filler was physically dispersed within the polymer matrix. As a consequence of weak interfacial adhesion, critical mechanical properties were drastically reduced. Tensile strength decreased from 63.2 MPa for pure PLA to 20.2 MPa for sample P30 (a 68% reduction), while impact strength dropped from 22.8 kJ/m^2^ to 6.2 kJ/m^2^ (a 73% reduction), confirming that the filler acted as a stress concentrator.

Despite the decrease in strength, the filler exhibited a stiffening effect, leading to an increase in Young’s modulus from 2131 MPa to 2408 MPa (a 13% increase for P20) and in the storage modulus (E′) at 30 °C from 2592 MPa to 3048 MPa (an 18% increase for P20). At 30 wt% filler content, particle agglomeration was observed, resulting in a decrease in material density from 1.240 g/cm^3^ to 1.193 g/cm^3^ and a reduction in stiffness.

The rheological and thermal properties of the composites were significantly affected. The addition of yeast led to more than a twofold increase in the melt flow rate (MFR) from 18.8 g/10 min to 39.8 g/10 min, resulting from the synergistic effects of plasticization and partial PLA degradation during processing. DMA and DSC analyses confirmed the plasticizing effect, manifested by a systematic decrease in the glass transition temperature (T_g_) from 71.8 °C to 68.8 °C and from 60.0 °C to 55.6 °C, respectively. The filler also exhibited a dual effect on crystallization; it acted as a nucleating agent at low concentrations, but at higher contents it inhibited crystallization, resulting in materials with a predominantly amorphous structure.

The thermal stability of the composites, evaluated by TGA, was significantly reduced; the temperature corresponding to 5% mass loss (T_d_) decreased from 329.3 °C for pure PLA to 266.8 °C for P30, considerably narrowing the processing window. At the same time, the effect on biodegradation under industrial composting conditions was nonlinear: the addition of 5 wt% yeast slowed the process (mass loss of 10.9% compared to 25.5% for PLA), whereas at 30 wt% filler content degradation was accelerated, reaching a mass loss of 27.5%.

It should be emphasized that although some composite properties are lower than those of pure PLA, this does not disqualify the developed material for practical applications. In many industrial sectors, such as single-use packaging, agricultural components, or short-lifecycle biodegradable products, mechanical requirements are moderate, while low cost, waste utilization, and biodegradability are of greater importance. In such applications, PLA/yeast composites may prove more competitive than pure PLA, combining favorable processing properties with ecological and economic benefits.

Waste yeast biomass can serve as a low-cost, biodegradable filler for PLA. While its incorporation without modification leads to decreased mechanical and thermal properties, it simultaneously improves processability and may accelerate material biodegradation at high filler loadings. Further research should focus on enhancing interfacial adhesion to achieve composites with a more balanced property profile.

In conclusion, the developed material should be considered promising—not as a direct substitute for PLA in high-strength applications, but as a more cost-effective, sustainable, and processable alternative for market segments where environmental aspects and the potential for biological recycling are important. The use of brewer’s yeast as a biofiller offers a unique balance of environmental and economic advantages. As a secondary raw material derived from the brewing industry, yeast biomass is widely available at low cost, supporting circular-economy resource utilization and reducing the reliance on virgin biopolymer feedstocks. The increased melt flowability observed in the composites indicates improved processability relative to neat PLA, which can translate into reduced energy demand and faster cycle times during injection molding of disposable products. Additionally, the biological origin and hydrophilic nature of yeast enhance water uptake and facilitate hydrolysis, which may accelerate biodegradation under industrial composting conditions—an asset for short-lifecycle applications. These benefits differentiate yeast favorably from many conventional inorganic fillers that do not contribute to end-of-life degradation.

From an application standpoint, the observed increase in stiffness (up to ~13% for P20) may be advantageous in products where dimensional stability, shape retention, and reduced elastic deformation are required. Examples include short-lifecycle rigid packaging (e.g., food containers, disposable trays), horticultural elements (seedling pots, plant clips), and consumer items produced by injection molding that must maintain form under moderate loads. In such cases, the slight reinforcement effect introduced by yeast particles could improve functional performance while simultaneously reducing cost and increasing bio-based content.

Conversely, higher stiffness accompanied by a substantial decrease in tensile and impact strength would be disadvantageous in applications requiring resistance to dynamic or cyclic loading. Products such as clips, fasteners, or snap-fit components rely on controlled flexibility and toughness, and excessive brittleness would significantly reduce service life. Therefore, while the stiffness enhancement itself is beneficial in targeted applications, the concurrent loss of ductility and impact resistance limits the suitability of these composites to low-stress, single-use or semi-rigid products where sustainability and biodegradation potential are prioritized over mechanical durability.

## Figures and Tables

**Table 1:** Mechanical properties of the tested materials determined from the static tensile test.

Sample	σ_M_ [MPa]	ε_M_ [%]	σ_B_ [MPa]	ε_B_ [%]	E [MPa]
P	63.2 ± 0.7	3.9 ± 0.2	60.5 ± 3.9	4.6 ± 0.8	2131 ± 115
P5	55.6 ± 0.9	3.1 ± 0.1	51.1 ± 1.4	4.2 ± 0.6	2334 ± 36
P10	42.6 ± 1.4	2.4 ± 0.1	37.4 ± 2.8	4.1 ± 1.0	2363 ± 74
P20	28.9 ± 0.4	1.7 ± 0.1	23.6 ± 1.4	4.9 ± 1.2	2408 ± 50
P30	20.2 ± 0.3	1.5 ± 0.2	17.6 ± 0.6	4.9 ± 1.0	2266 ± 100

**Table 2:** Thermomechanical parameters of the tested materials.

Sample	E′_30_ [MPa]	E′_50_ [MPa]	E′_80_ [MPa]	E′_100_ [MPa]	E′_140_ [MPa]	T_gDMA_ [°C]	Tan δ
P	2592	2545	7.2	4.5	93.8	71.8	2.412
P5	2704	2631	7.9	8.3	104.2	71.2	2.254
P10	2835	2751	8.6	9.5	103	70.1	2.079
P20	3048	2830	11.8	9.3	116.6	69.4	1.738
P30	2898	2717	15.7	11.1	133.2	68.8	1.488

**Table 3:** Thermal parameters of tested materials.

Sample	TG			DSC					
	T_d_ [°C]	T_max_ [°C]	R [%]	T_g_ [°C]	T_cc_ [°C]	ΔH_cc_ [J/g]	T_m_ [°C]	ΔH_m_ [J/g]	X_c_ [%]
P	329.3	376.1	0.0	60.0	121.2	21.2	149.6	22.9	1.8
P5	333.3	382.7	0.4	58.7	113.4	27.6	148.1	26.3	0
P10	327.3	381.5	1.0	57.8	115.4	27.2	147.9	25.7	0
P20	286	375.8	1.6	56.6	127.4	14.7	150.6	15.5	0
P30	266.8	372.8	2.8	55.6	126.1	15.4	149.3	14.9	0

## Data Availability

The original contributions presented in this study are included in the article. Further inquiries can be directed to the corresponding authors.
